# Functional genomics tools applied to plant metabolism: a survey on plant respiration, its connections and the annotation of complex gene functions

**DOI:** 10.3389/fpls.2012.00210

**Published:** 2012-09-06

**Authors:** Wagner L. Araújo, Adriano Nunes-Nesi, Thomas C. R. Williams

**Affiliations:** ^1^Departamento de Biologia Vegetal, Universidade Federal de Viçosa, ViçosaBrazil; ^2^Max-Planck Partner Group, Departamento de Biologia Vegetal, Universidade Federal de Viçosa, ViçosaBrazil

**Keywords:** functional genomics, systems biology, plant respiration, omics, data integration, modeling approaches

## Abstract

The application of post-genomic techniques in plant respiration studies has greatly improved our ability to assign functions to gene products. In addition it has also revealed previously unappreciated interactions between distal elements of metabolism. Such results have reinforced the need to consider plant respiratory metabolism as part of a complex network and making sense of such interactions will ultimately require the construction of predictive and mechanistic models. Transcriptomics, proteomics, metabolomics, and the quantification of metabolic flux will be of great value in creating such models both by facilitating the annotation of complex gene function, determining their structure and by furnishing the quantitative data required to test them. In this review, we highlight how these experimental approaches have contributed to our current understanding of plant respiratory metabolism and its interplay with associated process (e.g., photosynthesis, photorespiration, and nitrogen metabolism). We also discuss how data from these techniques may be integrated, with the ultimate aim of identifying mechanisms that control and regulate plant respiration and discovering novel gene functions with potential biotechnological implications.

## INTRODUCTION

Metabolism represents one of the most important and most likely the best characterized network within biological systems. From pioneering studies defining key metabolic pathways, subsequent decades of enzymology characterizing the catalytic and regulatory properties of enzymes, through to more recent genetic studies of metabolism, there is an unprecedented density of both mechanistic and descriptive data relating to metabolic behavior. Advances in the understanding of metabolic regulation and control, however, still suffer from insufficient research concerning the mode of operation of metabolic networks, and plant respiratory metabolism provides an illustrative example of this problem. Due to the crucial roles of mitochondria in meeting the energy demands of the cell whilst simultaneously being involved in amino acid metabolism and photorespiration, precise control of mitochondrial metabolism and function is crucial for cellular homeostasis, and unsurprisingly mitochondrial dysfunction leads to diverse metabolic and phenotypic consequences (Nunes-Nesi et al., 2011). However, despite the fact that the major respiratory pathways in plants were elucidated decades ago (Beevers, 1961), our current knowledge about their regulation and control, and how this relates to whole plant physiology, is still limited (Fernie et al., 2004). Unsurprisingly then, considerable research effort is being devoted to elucidating the metabolic basis of the regulation of the tricarboxylic acid (TCA) cycle and the mitochondrial electron transport chain (Sweetlove et al., 2007, 2010; Fahnenstich et al., 2008; Fukushima et al., 2009; Araújo et al., 2010; Tomaz et al., 2010; Zell et al., 2010) as well as their interactions with photosynthesis (Nunes-Nesi et al., 2007, 2011), photorespiration (Bauwe et al., 2010, 2012), and nitrate assimilation (Foyer et al., 2011).

Given the complex patterns of regulation and the interactions between respiratory metabolism and other processes uncovered by such studies, it is likely that only through the use of model-based approaches we will be able to understand how the individual components of metabolism (e.g., enzymes and metabolites) work together to produce a functioning system, and in this way truly understand the function of individual components of that system. In this context systems biology offers the promise of a better understanding of respiratory metabolism and its role within the metabolic network.

Systems biology aims to understand how populations of molecules, cells, and organisms interact to give rise to complex biological processes, including cell division, growth, development, metabolism, and behavioral and ecological patterns (Karsenti, 2012). Although initially applied exclusively to mathematical modeling strategies (Edwards and Palsson, 1999) systems biology may now incorporate data from the functional genomics tools of transcriptomics, proteomics, and metabolomics as well as genomics (Somerville et al., 2004; Baginsky and Fernie, 2007), and has become a truly interdisciplinary area of research, some basic and applied aspects of which have been expertly reviewed elsewhere (Ideker et al., 2001; Oltvai and Barabási, 2002; Kitano, 2004; Somerville et al., 2004; Breitling, 2010). Whilst an accurate understanding of metabolism as a system requires far more than enumeration of its components (Kleessen et al., 2012), it is nonetheless the case that the development of mathematical models of plant metabolism in general will require accurate gene annotations, functional characterization of enzymes, and the acquisition and statistical analysis of quantitative data.

In the case of plant respiratory metabolism, whilst the core components, including enzymes of the TCA cycle and components of the mitochondrial electron transport chain, have been identified and characterized in numerous species (Millar et al., 2011; Araújo et al., 2012a), how they are regulated in response to changing environmental conditions and developmental programs remains less clear, and extent to which respiratory metabolism interacts with other parts of the metabolic network is only now beginning to emerge (Balmer et al., 2004; Sweetlove et al., 2006; Timm et al., 2008, 2011; Fukushima et al., 2009; Tomaz et al., 2010; Foyer et al., 2011). Moreover, the functions of more peripheral components of plant respiratory metabolism remain to be investigated. Genes for many of the mitochondrial transporters that are hypothesized to exist have yet to be identified, or have only been assigned on the basis of homology with non-plant species (Linka and Weber, 2010; Palmieri et al., 2011), whilst it is only recently that several alternative electron donors to the mitochondrial electron transport chain have been characterized (Ishizaki et al., 2005, 2006; Araújo et al., 2010). Although here we focus mainly on function and interactions of respiratory enzymes, it is important to note that the construction of predictive models of mitochondrial metabolism will also require a better understanding of electron transport processes. Thus, both structural biology and measurements of electron transport rates remain exciting topics for further study.

**FIGURE 1:**
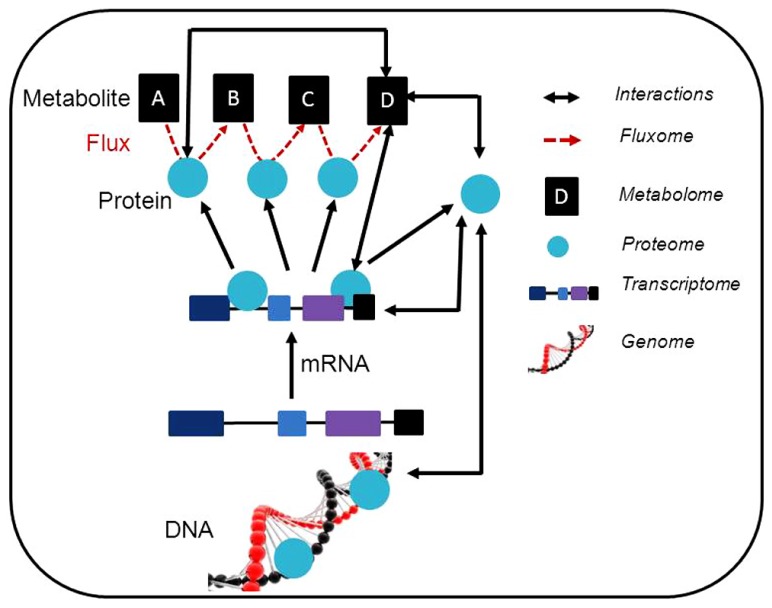
**Schematic representation of a synergetic integration of multiple omics approaches.** Within individual experiments, a range of data is generated. This data can be associated with different levels of omics and the possible interactions are illustrated. The usage of modeling approaches linking the interactions between the results obtained using different post-genomic techniques is expected. Additionally, the data obtained can be deposited in specific database contributing to the construction of general prediction databases (e.g. coexpression database). This also allows the generation of further testable hypothesis and to adjust such hypothesis for individual experiments.

The availability of comprehensive plant genome information, coupled with the integration of large-scale unbiased molecular profiling technologies such as whole-genome microarrays, quantitative proteomics, and metabolite profiling (**Table 1**; **Figure 1**) has increased our ability to annotate gene functions within plant respiratory metabolism, obtain quantitative data and uncover unanticipated relationships between respiration and other cellular processes such as photosynthesis, photorespiration, redox regulation, and signaling (Sweetlove et al., 2002; Heazlewood et al., 2004; Dutilleul et al., 2005; Urbanczyk-Wochniak et al., 2006). This information will ultimately facilitate the application of systems biology to plant respiratory metabolism (Fernie, 2012) and progress in these areas as they relate to research on plant respiration and the annotation of gene function will be discussed in the following sections.

**Table 1:** Omics fields associated with illustrative and available *Arabidopsis* resources on web.

Layers	Instances	Resources	Web
Phenome	Natural variations	NASC	http://arabidopsis.org.uk/home.html	
		ABRC	http://abrc.osu.edu/
	Mutant lines	TILLING	http://tilling.fhcrc.org/
		T-DNA tag line	http://signal.salk.edu/tabout.html
Metabolome	Metabolite profiles	Golm Metabolome Database	http://gmd.mpimp-golm.mpg.de/
		PRIMe	http://prime.psc.riken.jp/
	Metabolic maps	Reactome	http://www.reactome.org/ReactomeGWT/entrypoint.html
		PMN AraCyc	http://plantcyc.org/
Proteome	Proteome	RIPP-DB	http://phosphoproteome.psc.database.riken.jp/
		PPDB	http://ppdb.tc.cornell.edu/
		PhosPhAt	http://phosphat.mpimp-golm.mpg.de/
	Subcellular localization	PODB2	http://podb.nibb.ac.jp/Organellome/
		SUBAII	http://suba.plantenergy.uwa.edu.au/
		NASC proteome database	http://proteomics.arabidopsis.info/
Transcriptome	Full-length cDNA clones	RAFL clones	http://www.brc.riken.jp/lab/epd/catalog/cdnaclone.html
	ESTs	RARGE	http://rarge.psc.riken.jp/
	Expression profiles	AtGenExpress	http://www.arabidopsis.org/portals/expression/microarray/ATGenExpress.jsp
		Genevestigator	https://www.genevestigator.com/gv/
	Co-expression network	ATTEDII	http://atted.jp/
Genome	Sequence	TAIR	http://www.arabidopsis.org/
	Annotation		

## APPLYING TRANSCRIPTOMICS TO THE STUDY OF MITOCHONDRIAL FUNCTION

Determining the complete transcriptional capability of the cell, including large and small RNAs, novel transcripts from unannotated genes, splicing isoforms, and gene-fusion transcripts, serves as the foundation for a comprehensive study of the transcriptome. Recent advances in DNA sequencing technology have greatly increased both sequencing scale and throughput and led to the development of RNA-Seq (Liu et al., 2011; Martin and Wang, 2011), a technique capable of complementing microarray-based transcriptomics. In recent years transcriptomic techniques have been extensively used to reveal interactions between plant respiratory metabolism and other important processes such as seed germination (Howell et al., 2009), stress tolerance (Giraud et al., 2008; Lehmann et al., 2009; Meyer et al., 2009), hypoxia (Narsai et al., 2009), and the operation of the circadian clock (Fukushima et al., 2009).

The combination of a comprehensive list of putatively mitochondrially expressed genes with a large number of publicly available stress-related microarray datasets was used to define the mitochondrial stress response in *Arabidopsis *(van Aken et al., 2009). This complex analysis indicated that the mitochondrial stress response extends far beyond the alternative electron transport chain components, with, most prominently, mitochondrial substrate carrier proteins and heat shock proteins also showing extensive stress responsiveness. It is important to note, however, that many of the stress-responsive genes discovered by this approach are of unknown function and this indicates an attractive avenue for research aiming to identify new mitochondrial functions and targets for engineering of stress tolerance.

Transcriptomic studies can also provide details of expression patterns during plant development. Recent work indicated that BCS1, a likely AAA-ATPase, displayed similar levels of expression throughout development (Duncan et al., 2011), despite the fact that previous studies have shown that this gene responds to various stresses (van Aken et al., 2009). Based on these patterns of expression the authors propose that the protein encoded by this gene may be involved in protein repair in response to stress (Xu et al., 2011). This study also indicated that the transcript abundance for genes encoding outer and inner membrane mitochondrial proteins was maximal during seed germination, and identified a subset of genes that were highly expressed in root tissues (Duncan et al., 2011).

Responses to stress conditions, including the effect of absence of *alternative oxidase1a* (*aox1a*) expression in *Arabidopsis*, has also been investigated using transcriptomics. Such plants, when grown in moderate light under drought conditions, displayed 10-fold increases in leaf anthocyanin levels, together with alterations in photosynthetic efficiency, increased superoxide radical production, and reduced root growth (Giraud et al., 2008). Furthermore, microarray and quantitative reverse transcription polymerase chain reaction (qRT-PCR) analysis indicated that even under normal growth conditions genes normally induced under stress conditions were expressed (Giraud et al., 2008), including those involved in defense against reactive oxygen species (ROS) and in stress signaling. This study was incorporated into a recent meta-analysis of transcriptomic studies that aimed to uncover targets of mitochondrial retrograde signaling (Schwarzländer et al., 2012), and construct a model for how mitochondrial dysfunction may affect nuclear gene expression. Transcripts encoding proteins involved in photosynthesis, protein synthesis, and plant–pathogen interactions were revealed as the major targets of retrograde regulation.

A combination of transcript and metabolite profiling methods has been used to investigate the molecular and physiological responses following root hypoxia caused by flooding in gray poplar (*Populus* × *canescens*; Kreuzwieser et al., 2009). Interestingly, changes in metabolite levels occurred in both roots and leaves, whilst changes in transcript abundance were restricted to the roots, the actual site of hypoxia. The general pattern of metabolite and transcript abundance suggests that the response to hypoxia comprises both the repression of energy demanding processes such as cell wall biosynthesis, and the reconfiguration of carbohydrate and nitrogen metabolism to ensure sufficient substrate supply during long periods of root hypoxia (Kreuzwieser et al., 2009).

## PROTEOMIC APPROACHES FOR THE DISCOVERY OF GENE FUNCTION IN PLANTS

Proteomic approaches have been used to generate great insights into plant metabolism in general and into mitochondrial respiratory metabolism in particular. Recently, the utilization of multiple fluorescent dyes in single gels (DIGE) has renewed the use of 2-D gels for quantitative comparisons of the mitochondrial proteome. Studies using DIGE have investigated knockout mutants for carbonic anhydrase-like proteins (Perales et al., 2005), complex I (Meyer et al., 2009), and malate dehydrogenase (Tomaz et al., 2010), as well as differences between tissues (Lee et al., 2008), the impact of rotenone (Garmier et al., 2008), and changes associated with the diurnal cycle (Lee et al., 2010). Shotgun proteomic techniques utilizing Liquid chromatography coupled tandem mass spectrometry (LCMS/MS) of trypsin-digested samples, without the use of gels, have also been used for in-depth studies of the mitochondrial proteome in *Arabidopsis *(Brugiere et al., 2004; Heazlewood et al., 2004) and rice (Heazlewood et al., 2003b; Huang et al., 2009). These studies greatly increased the number of proteins identified within plant mitochondrial isolates that are below the level of detection by techniques based on gel separation and staining.

The mitochondrial proteome of *Arabidopsis* in particular has been extensively analyzed by both gel-based and gel-free strategies (Kruft et al., 2001; Millar et al., 2001; Heazlewood et al., 2004; Lee et al., 2008, 2011), and may contain as many as 2000–3000 different proteins, each of which may be subject to post-translational modification (Millar et al., 2005; Taylor et al., 2011). Recent proteomic studies have been able to identify more than 500 proteins, including subunits of mitochondrial respiratory complexes, supercomplexes, phosphorylated proteins, and oxidized proteins (Millar et al., 2005; Klodmann and Braun, 2011). However, even these identified proteins have not all been functionally characterized. Moreover, targeting prediction software tools assign more than 1,500 proteins encoded by the *Arabidopsis* genome to this subcellular compartment and it therefore seems likely that the function of most mitochondrial proteins, especially those of low abundance and/or high hydrophobicity (Klodmann and Braun, 2011), remains to be discovered.

In addition to analysis of the mitochondrial proteome as a whole, proteomic approaches have been used extensively to dissect mitochondrial protein complexes, in particular complex I of the respiratory electron transport chain (Leterme and Boutry, 1993; Herz et al., 1994; Klodmann and Braun, 2011), and the importance of this characterization using different proteomic approaches has recently been expertly reviewed (Klodmann and Braun, 2011). These methods have revealed that the complex I in plants has an extremely sophisticated configuration and although some of the postulated functions of the characterized extra subunits remain to be fully investigated, proteomic approaches have already contributed significantly to our current understanding of this complex (Heazlewood et al., 2003a; Cardol et al., 2004; Klodmann et al., 2010, 2011). For example, recent work has revealed that plant complex I is especially large and includes 15 extra unique subunits. Five of these subunits represent proteins resembling carbonic anhydrases and one represents a L-galactono-1,4-lactone dehydrogenase (GLDH), introducing side activities to plant complex I, while all the remaining nine subunits are rather small (7–12 kDa) and of so far unknown function (Klodmann et al., 2010; Klodmann and Braun, 2011).

Many mitochondrial proteins in fact form part of protein complexes and it has recently been shown that the formation of supercomplexes, “complexes of complexes,” could have a regulatory function in guiding electrons through alternative respiratory pathways, particularly under variations in oxygen levels (Ramírez-Aguilar et al., 2011). The extent to which this phenomenon occurs is currently unclear though as only the most abundant complexes have so far been functionally characterized.

Proteomics may also help to provide details of the mechanisms that regulate mitochondrial metabolism, and recent studies have identified potential thioredoxin-linked proteins in mitochondria isolated from autotrophic and heterotrophic plant tissues (Balmer et al., 2004; Martí et al., 2009). Fifty mitochondrial thioredoxin-linked proteins were identified including six TCA cycle enzymes [aconitase, succinyl-CoA ligase, isocitrate, malate, pyruvate, and succinate dehydrogenases (SDH); Balmer et al., 2004]. These findings suggest mitochondrial enzymes as potential targets for redox regulation through thioredoxins. However, despite the fact that these studies, amongst others, indicate that by sensing redox state thioredoxins enable mitochondria to communicate with other organelles in photosynthetic tissues, considerable experimental effort is still required to provide functional details.

## INSIGHTS INTO PLANT RESPIRATORY METABOLISM OBTAINED FROM METABOLITE PROFILING

The term metabolome can be defined as the total small-molecule complement of a cell, and metabolomics is therefore the study of all small molecules or metabolites presents in a cell or organism (Oliver et al., 1998; Tweeddale et al., 1998). Although no single analytical system is ever likely to cover the whole metabolome technological developments have considerably extended our ability to analyze complex biological systems, facilitating the simultaneous detection of different compound classes with diverse chemical properties (Osorio et al., 2012). Gas chromatography coupled to mass spectrometry (GC-MS) is a versatile and widely applied technique in modern metabolomic studies and allows the identification and quantification of a relatively broad range of compounds, including organic and amino acids, sugars, sugar alcohols, phosphorylated intermediates, and lipophilic compounds (Fiehn et al., 2000; Roessner et al., 2001; Weckwerth, 2003; Lisec et al., 2006; Schauer et al., 2006), resulting in fairly comprehensive coverage of the central pathways of primary metabolism. Metabolite profiling using LC-MS and capillary electrophoresis-MS can complement the analysis by GC-MS for certain classes of metabolite. Of particular note, tandem MS methods for the quantification of phosphorylated sugars (Lunn et al., 2006; Luo et al., 2007; Hasunuma et al., 2010), Calvin–Benson cycle intermediates (Arrivault et al., 2009) and cell wall precursors (Alonso et al., 2010), which are difficult to analyze by GC-MS, have recently been developed.

Due in part to these technological advances application of metabolite profiling to plant biology is becoming increasingly common, including diagnostic and descriptive analyses of metabolic responses to genetic and/or environmental perturbations, the use in annotation of gene function and in systems biology (Schauer and Fernie, 2006; Guy et al., 2008; Fernie and Schauer, 2009). Recently, metabolite profiling has, in particular, been used to generate significant insight into plant respiratory metabolism and its interactions with photosynthesis. Despite recognition of the importance of mitochondrial metabolism our understanding of the complex pathways through which organic acids are metabolized as well as how these pathways are regulated *in vivo* remains far from complete (Fernie et al., 2004; Sweetlove et al., 2007; Fait et al., 2008; Møller and Sweetlove, 2010). As part of attempts to remedy this issue, the operation and role of the TCA cycle in illuminated leaves has received particular interest (Krömer, 1995; Raghavendra and Padmasree, 2003; Noguchi and Yoshida, 2008; Nunes-Nesi et al., 2011). The precise mode of operation of the TCA cycle in the light remains somewhat obscure, in part because whilst TCA cycle flux in the light is reduced through inhibition of pyruvate dehydrogenase carbon skeletons deriving from TCA cycle intermediates are still required for nitrogen assimilation. In attempt to address this apparent contradiction, a process of systematic suppression of TCA cycle enzymes through reverse genetics was undertaken. Metabolite profiling of the transgenic lines generated through this process indicated the importance of the TCA cycle in metabolism in illuminated leaves (Nunes-Nesi et al., 2008, 2011). However, these experiments have also revealed a surprising complexity in the response to these manipulations, with suppression of some enzymes leading to increased photosynthesis (Carrari et al., 2003; Nunes-Nesi et al., 2005; Araújo et al., 2011b), others leading to decreased photosynthesis (Nunes-Nesi et al., 2007) and yet others having no effect (Studart-Guimarães et al., 2007; Sienkiewicz-Porzucek et al., 2008, 2010; Sulpice et al., 2010a; Araújo et al., 2012b). Several of the more recent studies from this investigation, highlighting the importance of information obtained from metabolite profiling experiments, are discussed here.

Antisense inhibition of the 2-oxoglutarate dehydrogenase (OGDH) complex in tomato led to alterations in whole plant development that were linked to reductions in total amino acid and nitrate pools despite unaltered photosynthesis (Araújo et al., 2012b). These results clearly imply that OGDH plays an important role in both the TCA cycle and nitrogen assimilation and suggest a novel role for this enzyme in whole plant development and fruit maturation. Furthermore, as was reported for succinyl-CoA ligase antisense plants (Studart-Guimarães et al., 2007), the OGDH antisense plants displayed increased GABA shunt flux, presumably in compensation for decreased succinate production via the TCA cycle (Araújo et al., 2012b). However, it is important to stress that such a compensatory response also caused significant shifts in cellular pools of amino acids and nitrate that were detected by metabolite profiling.

Antisense inhibition of the iron–sulfur subunit of the SDH in tomato plants, on the other hand, resulted in increased photosynthesis and whole plant biomass via an organic acid-mediated effect on stomatal aperture. These results contrast with those obtained for antisense inhibition of fumarase, where decreased photosynthesis and biomass were observed (Nunes-Nesi et al., 2007; Araújo et al., 2011b). Furthermore, measurement of apoplastic organic acid levels in SDH and fumarase antisense plants, revealed a negative correlation between the levels of fumarate and stomatal conductance, though the influence of fumarate appears to be weaker than that of malate (Araújo et al., 2011b). These results provided strong evidence to support that modulation of malate and fumarate concentration can greatly influence stomatal function. Thus, metabolite profiling has aided in the identification of novel interactions between the TCA cycle and photosynthesis, stomatal function and nitrogen metabolism. Further work is clearly required in order to establish the regulatory mechanisms involved in such responses.

Protein degradation during plant development and substrate deficiency can be an important source of substrate for respiratory metabolism, however, our current understanding of the regulation of the classical and alternative pathways of respiration under these conditions is still limited. The combination of genetic approaches with GC-MS-based metabolite profiling has greatly improved our understanding of the complex metabolic interactions observed during dark-induced senescence (Ishizaki et al., 2005, 2006; Araújo et al., 2010, 2011a). These studies have demonstrated that during dark-induced senescence there is a significant accumulation of amino acids and TCA cycle intermediates. Moreover, it has also been demonstrated that both isovaleryl-CoA dehydrogenase and D-2-hydroxyglutarate dehydrogenase provide electrons to the plant ubiquinol pool via the electron transfer flavoprotein (ETF)-ETF:ubiquinone oxidoreductase (ETF/ETFQO) complex (Engqvist et al., 2009; Araújo et al., 2010). Given that the chlorophyll breakdown intermediate phytanoyl-CoA accumulates dramatically both in knockout mutants of the ETF/ETFQO complex and of isovaleryl-CoA dehydrogenase following growth in extended dark periods it was suggested that chlorophyll breakdown could be important for the supply of carbon and electrons during this process. However, metabolic analyses of phytanoyl-CoA 2-hydroxylase knockout mutants under the same extended darkness regime as previously used suggest that phytol and phytanoyl-CoA enzyme does not primarily function as a substrate of the ETF/ETFQO pathway (Araújo et al., 2011a). These studies also showed that these mutants were not compromised in their ability to withstand significant extension of the dark period but do accumulate phytanoyl-CoA and to a lesser extent 2-hydroxyglutarate as well as sharing some of the other metabolic features of mutants of the ETF/ETFQO complex following dark-induced senescence treatment. In summary, the results obtained through this work indicated that both isovaleryl-CoA dehydrogenase and 2-hydroxyglutarate dehydrogenase essentially account for the entire electron input via the ETF complex.

Plant metabolism is also reorganized under a range of different stress conditions including salt, cold, drought, and oxidative stress (Kaplan et al., 2004, 2007; Gong et al., 2005; Sanchez et al., 2008, 2012; Alet et al., 2012; Siahpoosh et al., 2012), allowing plants to continue to produce indispensable metabolites whilst preventing the accumulation of ROS. Metabolite profiling has proven a powerful tool to gain an overview of such reorganizations in response to stressful conditions (Shulaev et al., 2008). Menadione, a quinone which causes ROS generation from both mitochondrial and plastidal electron transport chains, has been used to study metabolic and transcriptomic responses to ROS in *Arabidopsis* roots (Lehmann et al., 2009). Detailed metabolic studies revealed a down-regulation of glycolysis and TCA cycle and the redirection of carbon from glycolysis to the oxidative pentose phosphate pathway in menadione-treated roots suggesting a reorganization of central carbon metabolism under oxidative stress conditions that was undone rapidly after the removal of menadione (Lehmann et al., 2012).

## CONTRIBUTIONS OF METABOLIC FLUX ANALYSIS TO OUR UNDERSTANDING OF PLANT RESPIRATION

Metabolic flux ultimately underpins both plant growth and development and for this reason experiments that provide insight into metabolic flux, as well as its regulation and control, can be valuable tools to both improve our understanding of biological systems and aid in the discovery of gene function. Feeding experiments using both ^13^C- and ^14^C-labeled precursors have been used extensively to delineate metabolic pathways, but may also be used to investigate and quantify metabolic flux. Labeling experiments with ^14^C offer high sensitivity and fractionation of labeled metabolites and biomass components can readily indicate the fate of metabolized radiolabel. Accordingly, feeding experiments with ^14^CO_2_ demonstrated the effect of reduced malate dehydrogenase activity on photosynthetic carbon assimilation in tomato leaves, and revealed that ascorbate feeding led to increased photosynthesis and altered assimilate partitioning in these transgenic lines (Nunes-Nesi et al., 2005). Quantifying the release of ^14^CO_2_ from the metabolism of different isotopomers of ^14^C-glucose can also provide information about the relative activity of different primary metabolic pathways (Nunes-Nesi et al., 2005). This method has been used extensively to identify changes in metabolic flux in plants with altered TCA cycle enzyme activities; for example, decreased carbon entry into the TCA cycle was identified in tomato plants with decreased SDH and OGDH activity (Araújo et al., 2011b, 2012b), which are in good agreement with measurements of respiration and the results of metabolite profiling experiments.

Labeling experiments with stable isotopes can be used for similar ends, however since detection of label incorporation is in these cases carried out by mass spectrometry or Nuclear Magnetic Resonance (NMR) spectroscopy it is possible to determine both enrichment and location of incorporation of label in individual metabolites without laborious chemical cleavage. This method was used to investigate the fate of ^13^C-labeled glutamate in leaves of tomato plants with decreased succinyl-CoA ligase, and revealed that TCA cycle flux was maintained in these plants by diversion of carbon through the GABA shunt (Studart-Guimarães et al., 2007). Additionally, *in vivo* NMR spectroscopy can be used to monitor such labeling experiments in real-time. For example, tracing the metabolism of ^13^C-pyruvate by mitochondria isolated from *Arabidopsis* plants with reduced manganese superoxide dismutase activity revealed decreased TCA cycle flux (Morgan et al., 2008), most likely as a result of decreased TCA cycle enzyme activity caused by oxidative damage.

Whilst the experiments described above may provide valuable qualitative information about metabolic flux, the number of fluxes that can be quantified using these approaches is typically quite limited. Steady state metabolic flux analysis (Wiechert, 2001; Ratcliffe and Shachar-Hill, 2006) on the other hand allows the absolute quantification of metabolic fluxes in medium size networks, permitting the degree to which a particular environmental condition or gene product controls or regulates multiple fluxes to be determined. In this way, steady state flux analysis has been able to provide important insights into the relationship between photosynthesis and respiration in developing seeds. Labeling experiments and metabolic modeling indicated that developing green embryos are able to decrease losses of fixed carbon during oil synthesis by both refixing CO_2_ released by respiration (Schwender et al., 2006), and reducing the need for TCA cycle flux through the use of photosynthesis to meet energy demands (Allen et al., 2009). Although these experiments effectively revealed a new function for RuBisCO, steady state analysis can also be used to reveal the effect of alterations in gene expression. This method has been widely applied for the discovery of gene function in microorganisms (Blank et al., 2005; Fischer and Sauer, 2005), and its feasibility in plants was demonstrated by experiments using *Arabidopsis* embryos (Lonien and Schwender, 2009) that revealed how flux is rerouted in embryos deficient in two plastidic pyruvate kinase isoforms.

The lack of wider adoption of steady state flux analysis as a tool in plant systems biology is in part due to the strict requirements for an experimental system that can obtain metabolic and isotopic steady state, which limits such experiments to tissue culture and isolated organs. Therefore, the so called instationary flux analysis (Wiechert and Nöh, 2005; Young et al., 2008) has the potential to allow quantification of metabolic flux in networks that do not reach isotopic steady state, by incorporating measurements of labeling dynamics and metabolite pool sizes into the modeling process. Recent work has demonstrated the feasibility of this approach for flux quantification in photoautotrophic organisms (Young et al., 2011), whilst the necessary technological approaches for carrying out this work in plants have already been developed (Arrivault et al., 2009; Hasunuma et al., 2010), suggesting that this method may be used in the discovery of gene function in higher plants in the relatively near future.

Whilst empirical determination of fluxes using labeling experiments can generate important biological insight, constraints based modeling (Price et al., 2003) can also be used to investigate the behavior of metabolic networks. The construction of genome scale metabolic models (Durot et al., 2009; Fell et al., 2010) and their analysis using flux balance analysis, provides both a way to exploit the information pertaining to metabolism contained within a sequenced genome and models now exist for several plant species including *Arabidopsis *(Poolman et al., 2009; Mintz-Oron et al., 2012), maize (Saha et al., 2011), sugarcane (de Oliveira Dal’Molin et al., 2010), and *Brassica napus *(Hay and Schwender, 2011). Recent work has highlighted the degree to which flux quantification using constraints based modeling agrees with empirical results (Williams et al., 2010; Beurton-Aimar et al., 2011; Chen et al., 2011), but perhaps a more important role of genome scale modeling is as a means to generate hypotheses regarding the capacities of large metabolic networks.

In this regard, flux balance analysis has already provided interesting insights into plant respiratory metabolism. For example, work using a model of* Arabidopsis* carbon metabolism revealed the large demand for energy made by cell maintenance processes (Poolman et al., 2009), whilst a study of metabolism in barley seeds (Grafahrend-Belau et al., 2009) indicated how fluxes in central carbon metabolism and the TCA cycle may be affected by the hypoxic conditions that can occur within the developing seed. Such models also provide an excellent means for the discovery of new functions for previously annotated genes though the prediction of the consequences of gene knockouts *in silico*. Such methods are expected to form an important part of methods for metabolic engineering in species with biotechnological importance. Indeed, models have already been created for the model algal species *Chlamydomonas reinhardtii* (Boyle and Morgan, 2009; Chang et al., 2011; de Oliveira Dal’Molin et al., 2011) that can be further used to explore the potential of microalgae for biofuel production.

## INTEGRATION OF PROFILING DATA: FROM DIAGNOSTICS TO SYSTEMS BIOLOGY

Whilst investigation of the transcriptome, proteome, and metabolome alone may be highly informative, cellular physiology and the regulation of plant respiratory metabolism is the result of complex interactions between transcripts, proteins, and metabolites. Bearing that in mind straightforward relationships between these components of the metabolic network are therefore not to be expected. Thus, despite the apparent easy understanding of simple relationships between changes in transcripts, proteins, metabolite, and downstream biological functions, quite often there is a major discordance. This apparent discrepancy can often be explained by quantitative analysis of the data sets and information about translational regulation and protein turnover. These incoherencies are not only due to technical issues but also related to the complexity and structure of metabolic networks. Accordingly, some priorities in metabolic research to reduce these problems have recently been proposed (Fernie and Stitt, 2012). For this reason recent research into plant metabolism has often included the usage of modeling and correlation based approaches, and interactions between the results obtained using different post-genomic techniques (**Figure 1** and **Table 1**) are expected.

Such studies have the ability to reveal previously unappreciated and complex interactions between transcripts, proteins, metabolites, and fluxes. In this way, a recent study was able to uncover a potential signaling role for leucine; a combination of extensive metabolite profiling and transcript profiling demonstrated a correlation between expression of a subset of transcripts and the levels of this amino acid (Hannah et al., 2010). An ability to simultaneously quantify components of the metabolic network can also provide information about the relationship between different components of central carbon metabolism and resource allocation. For example, by exploiting the natural variation present in different accessions of *Arabidopsis *(Sulpice et al., 2009, 2010b), it was possible to detect correlations between enzyme activities, metabolite abundances, and biomass. These studies indicate the presence of a strong negative correlation between starch and biomass, whilst the relative proportion of protein invested in enzymes is positively correlated to biomass accumulation. The relationship between transcripts, metabolites, and fluxes in plant respiratory metabolism was investigated in *Arabidopsis* cell suspension cultures subjected to menadione treatment (Baxter et al., 2007). The combinatory analysis of these parameters identified a transient reconfiguration of metabolism, involving down-regulation of the TCA cycle and amino acid biosynthesis, apparently working to avoid wasting energy under conditions of oxidative stress. Such methods may also have biotechnological applications. For example, metabolite:transcript correlations identified in potato tubers have revealed target genes for metabolic engineering of sucrose metabolism (Urbanczyk-Wochniak et al., 2003).

Whilst the unbiased nature of such approaches makes them excellent discovery tools, their capacity to improve our understanding of the regulation and control of respiratory metabolism could be further improved by the use of a mechanistic framework that can relate the components of respiratory metabolism and their properties to the functions of this system. In this regard, a strategy recently employed in yeast linking transcriptional regulation to metabolic fluxes (Moxley et al., 2009) may facilitate the integration of data from multiple post-genomic platforms to improve our understanding of plant respiratory metabolism. Whilst direct mRNA measurements were only poorly correlated with metabolic flux, the incorporation of a parameter representing regulation of enzyme activity by metabolite abundance greatly improved this correlation and the resulting model could be used successfully to predict flux changes occurring in amino acid metabolism in yeast. Genome scale metabolic models also provide a promising means via which such integration can take place (Durot et al., 2009; Poolman et al., 2009; Fell et al., 2010; Mintz-Oron et al., 2012). Since such models are based on annotated genome information, transcript profiling, and proteomic data which are already linked to specific genes can potentially be incorporated and used to constrain the behavior of the network. Such an approach has been shown to be useful in microorganisms (Covert and Palsson, 2002) and might also be successfully applied in plants.

Integration may also take place through the use of kinetic models of metabolism (Schallau and Junker, 2010; Rohwer, 2012). Such models are inevitably on a smaller scale than genome scale models, but compensate by providing a mechanistic link between the kinetic and regulatory properties of enzymes and the behavior of the system under study. This approach was used to study metabolism of the aspartate family of amino acids in *Arabidopsis* (Curien et al., 2009), and revealed how the allosteric properties of enzymes in this pathway permit fluxes in different branches to vary independently of one another. An additional advantage of such models is that once constructed they immediately allow the application of metabolic control analysis, and this has been exploited to investigate the distribution of control over sucrose cycling amongst the enzymes of sucrose synthesis in sugarcane (Rohwer and Botha, 2001; Uys et al., 2007). Whilst the mitochondrial TCA cycle in plants has been the subject of structural modeling (Steuer et al., 2007) and included in models of wider scope (Leduc et al., 2006), a detailed kinetic model based on experimental data has yet to be produced. Profiling techniques could aid in the provision of data for the construction of such a model, but more importantly, a kinetic model of the plant TCA cycle would provide a medium through which the results of profiling experiments could be better understood.

Overall, whilst post-genomic technologies have already aided our understanding of respiratory metabolism at a systems level through additions to the “parts list” of the metabolic network (Sweetlove and Fernie, 2005), they are poised to become more useful still through the discovery of new interactions and the provision of the quantitative data required for the construction of predictive models of metabolism. The further development and combination of many analytical techniques (**Figure 1**) will additionally allow a fuller description of the metabolic status of a plant. When this is achieved, global analyses of RNA, protein, and metabolites will allow us to obtain a full picture of the complexity of the system under study. Although the application of these techniques requires substantial financial investment, it is likely to bring returns in the form of an improved ability to carry out rational engineering of the plant metabolic network.

## Conflict of Interest Statement

The authors declare that the research was conducted in the absence of any commercial or financial relationships that could be construed as a potential conflict of interest.
